# Changes in glucagon‐like peptide 1 and 2 levels in people with obesity after a diet‐induced weight‐loss intervention are related to a specific microbiota signature: A prospective cohort study

**DOI:** 10.1002/ctm2.575

**Published:** 2021-11-06

**Authors:** M‐Mar Rodríguez‐Peña, Brenno Astiarraga, Jesus Seco, Victoria Ceperuelo‐Mallafré, Adrià Caballé, Vicente Pérez‐Brocal, Camille Stephan‐Otto Attolini, Andrés Moya, Gemma Llauradó, Ana Megía, Silvia Pellitero, Núria Vilarrasa, Sonia Fernández‐Veledo, Joan Vendrell

**Affiliations:** ^1^ Endocrinology and Nutrition Service Hospital Universitari de Tarragona Joan XXIII Institut d'Investigació Sanitària Pere Virgili Tarragona Spain; ^2^ CIBER de Diabetes y Enfermedades Metabólicas Asociadas (CIBERDEM)‐Instituto de Salud Carlos III Madrid Spain; ^3^ Departament de Medicina i Cirurgia Universitat Rovira i Virgili Tarragona Spain; ^4^ Institute for Research in Biomedicine (IRB Barcelona) The Barcelona Institute of Science and Technology Barcelona Spain; ^5^ Department of Genomics and Health Foundation for the Promotion of Health and Biomedical Research of Valencia Region (FISABIO‐Public Health) Valencia Spain; ^6^ Biomedical Research Networking Centre for Epidemiology and Public Health (CIBERESP) Madrid Spain; ^7^ Spanish National Research Council (CSIC) Institute for Integrative Systems Biology (I2SysBio) University of Valencia Valencia Spain; ^8^ Department of Endocrinology and Nutrition Hospital del Mar Institut Hospital del Mar d'Investigacions Mèdiques (IMIM) Universitat Autònoma de Barcelona Barcelona Spain; ^9^ Endocrinology and Nutrition Department Institute Research and Hospital Germans Trias i Pujol Universitat Autònoma de Barcelona Barcelona Spain; ^10^ Department of Endocrinology and Nutrition L'Hospitalet de Llobregat Bellvitge University‐IDIBELL Barcelona Spain

Dear Editor,

Glucagon‐like peptide (GLP)‐1 and ‐2 are enteroendocrine hormones released postprandially to regulate glucose metabolism.^1^, ^2^ For reasons that are unclear, GLP‐1/2 secretion is impaired in obesity.^3^ The enteroendocrine system has been linked to gut microbiota,^4^ but whether this could be affected by obesity‐induced dysbiosis is unknown. Here, we investigated the relationship between GLP dynamics and gut microbiota in a prospective cohort of patients with obesity enrolled in a 6‐month restrictive diet interventional weight‐loss trial (trial registration number ISRCTN12973246), where effective weight loss (≥10%) was achieved at the end of the follow‐up. We determined the association between GLP secretion in response to nutrient ingestion and specific metagenomic signatures using taxonomic profiling and functional analysis.

The main characteristics of the cohort are described in Table S1. Patients showed improvement in glucose and lipid profiles at 6 months. Notably, fasting GLP‐1/2 levels were significantly lower after weight loss, with a trend for improved GLP response to a meal tolerance test (MTT). The intervention had no significant impact on intra‐community (alpha) diversity measured by species richness or evenness (Figure S1), in agreement with other reports.^5^, ^6^, ^7^ Nonetheless, a higher alpha diversity was observed in patients with lower plasma urate levels, lower systolic blood pressure, and lower weight (*p *= 0.008, 0.026, and 0.04, respectively), all related to a “healthy” metabolic status. No differences were found in inter‐community (beta) diversity, as estimated by the Bray–Curtis distance (*p* = 0.216), or when microbial populations at different taxonomic levels were compared before and after the intervention (Figure 1A). Remarkably, the unique patient identifier variable correlated with all tested principal components (Figure S2), pointing to individual variation as the main driver of the data variability. Accordingly, we rejected the hypothesis of microbial composition independence among pre‐ and post‐intervention observations from the same patient, which stresses the importance of accounting for a paired data setting in the downstream analysis (results obtained at family taxonomic level, Figure 1B).

**FIGURE 1:**
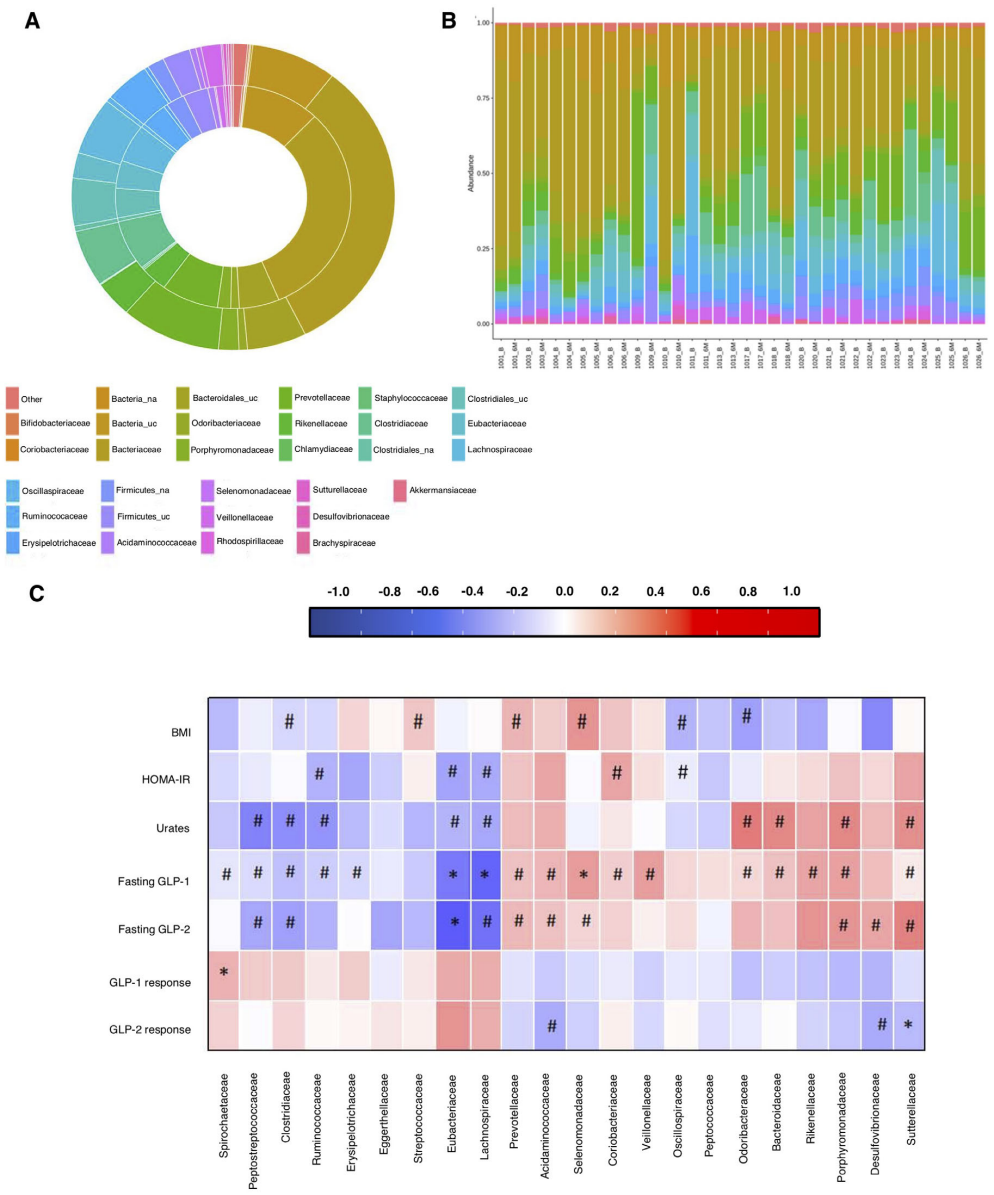
Taxonomy composition of the study population. (A) Comparison of relative abundances of different taxa at the family level between post‐intervention (outerchart) and basal (inner chart) faecal microbiota, showing no significant differences. (B) Individual variability between basal (B) and post‐intervention (6 M). The presence of different microorganisms is indicated by colours; the same colour indicates the same family. Microbial taxa are based on the family level. Significant differences were observed for each individual after diet intervention (*p*‐value < 0.05). The chi‐square test was used for analysis (*n* = 18 classified as basal (B) and post‐intervention (6 M)). (C) Associations of gut microbiota with clinical variables and intestinal hormones. Heatmap depicting Spearman's rank correlation coefficient of the relative abundances at the family level of different gut microbiota and clinical variables and incretin hormones in all individuals. For correlational studies, all gathered data (basal and 6 months) are included in the analysis. Adjusted *p*‐values: #*p*adj < 0.25; *padj < 0.05. Peptostreptococcaceae, Clostridiaceae, Ruminococcaceae, Oscillospiraceae, Eubacteriaceae, and Lachnospiraceae showed negative associations with BMI, HOMA‐IR index, and/or plasma urates. By contrast, Streptococcaceae, Prevotellaceae, Selenomonadaceae, Coriobacteriaceae, Bacteriodaceae, Rikenellaceae, Porphyromonadaceae, and Sutterellaceae showed positive associations with the afore mentioned parameters. Odoribacteriaceae showed a negative association with body mass index (BMI) and a positive association with plasma urates

Association analysis of clinical parameters and gut microbiota revealed several significant associations between different bacterial families and body mass index, HOMA‐IR, and/or plasma urates (Figure 1C).

Fasting GLP‐1/2 levels were inversely related to Spirochaetaceae, Peptostreptococcaceae, Clostridiaceae, Ruminococcaceae, Erysipelotrichaceae, Eubacteriaceae, and Lachnospiraceae, and positively related to Prevotellaceae, Acidaminococaeae, Selenomonadaceae, Coriobacteriaceae, Veillonelaceae, Odoribacteriaceae, Bacteriodaceae, Rikenellaceae, Porphyromonadaceae, Desulfovibrionaceae, and Sutterellaceae. Notably, we found a contrasting correlation pattern for GLP‐1/2 response in the MTT, as Spirochaetaceae showed a significant positive correlation with GLP‐1 response, whereas the GLP‐2 response was negatively correlated with Acidaminococcaceae, Desulfovibrionaceae, and Sutterellaceae (Figure 1C).

To address the statistical sparsity issues and the lack of homogeneously distributed variables among individuals (common to microbiome data analysis), we applied the FitZig mixture model,^8^ which allows for discrimination of taxonomic difference abundances between the study groups. Differential abundance analysis pre‐/post‐intervention revealed more than 40 species or genera with a significant variation in terms of fold‐change and statistical relevance when considered simultaneously (Figure S3). Abundance ranking analysis revealed noteworthy interindividual variations, suggesting that communities from the same individual were generally more similar to one and other than to those of other individuals, with individual variability more important than diet–responsive variation (Figure 1B). These results are in line with previous studies showing that human gut microbiota response to diet has a strong individuality and indicating that the effects of the diet per se as the main source of variability are markedly diminished, which is the opposite to that described in mice.^9^, ^10^

Most of the significantly increased taxa at follow‐up belonged to the Clostridiaceae family, specifically the genus *Clostridium* (Figure 2). Analysis of GLP‐1/2 dynamics revealed a positive association between *Mitsuokuella*_uc and fasting GLP‐1/2 levels, but a significant negative association between *Mitsuokuella*_uc and GLP‐2 response in the MTT. Contrastingly, *Clostridium* sp. CAG:75, CAG:230, and CAG:127 and *Lachnospiraceae bacterium* 5_1_63FAA (which all increased after the weight loss intervention) correlated negatively with fasting levels of GLP‐2 but positively with its response in the MTT. Of note, *Clostridium* sp. CAG:127 showed a significant positive correlation with GLP‐2 response. Similarly, *Clostridium* sp. CAG: 230 correlated negatively with fasting GLP‐1 but positively with GLP‐1 response in the MTT (Figure 2). Overall, the data indicate that fasting levels of GLPs are inversely related to their response to the MTT, with higher levels signalling poor responsiveness. Notably, this behaviour, which differed for GLP‐1 and GLP‐2 was, at least in part, associated with a specific microbiota signature.

**FIGURE 2:**
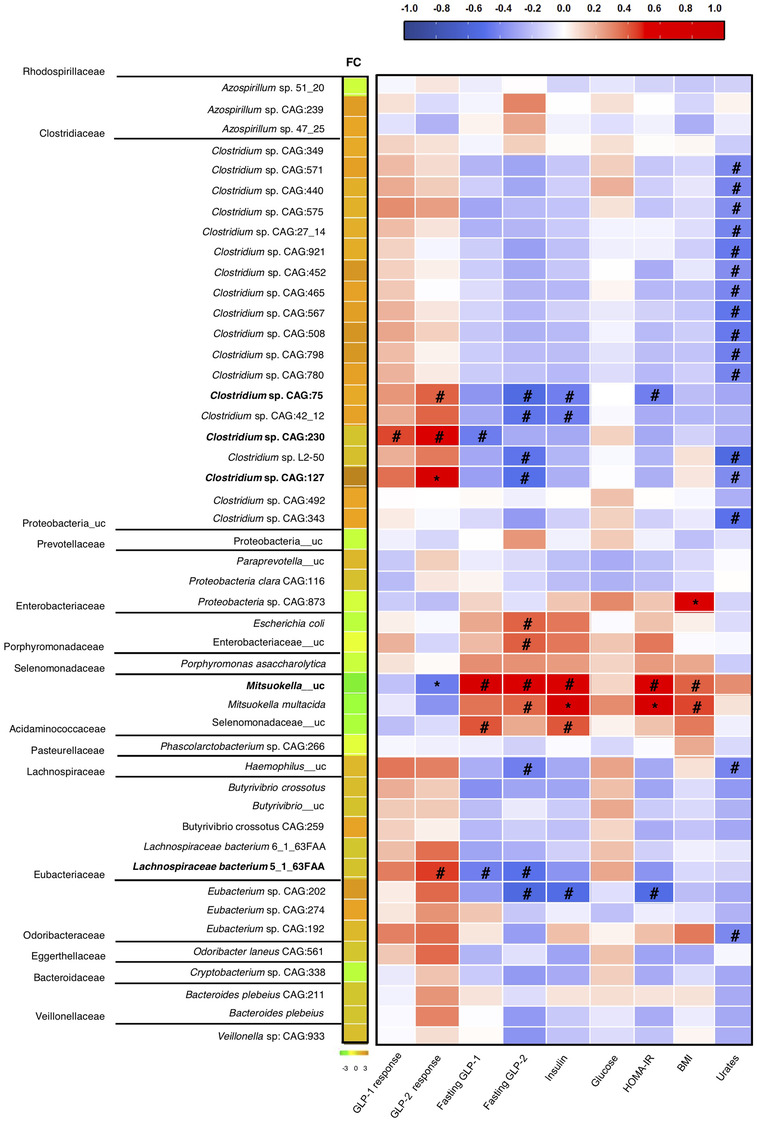
Diet‐induced microbiota changes and associations between species and variables related to metabolic health status. Log fold‐change (FC) and Spearman correlation analyses of the relative abundance at the species level of gut microbiota and clinical variables and incretin hormones in all individuals. For FC analysis, a zero‐inflated Gaussian mixture model (fitZig) from the metagenomeSeq R package was used, contrasting 6 M/basal. The subject factor in the patient identifier variable (IDPAT) is used as a batch effect, as the inter‐individual differences in the microbiota were greater than the changes caused by the diet. As a consequence, the IDPAT variable was introduced as an adjusting covariate in the model to investigate diet‐induced changes after the 6‐month weight‐loss program. Spearman correlation analysis revealed a negative correlation between most of the *Clostridium* species, *Hemophilus*_uc, and *Eubacterium* sp. CAG 192 and plasma urates, whereas *Clostridium* sp. CAG:75 and *Eubacterium* sp. CAG 202 were negatively correlated with HOMA‐IR index. *Proteobacteria* sp. CAG:873 and *Mitsuokella multocida*, showed a significant positive correlation with body mass index (BMI) and HOMA‐IR, respectively. All *p*adj < 0.005 in FC and #*p*adj < 0.25; *padj < 0.05 for Spearman correlation analyses

The study at functional gene level identified significant associations between the expression of non redundant genes aligned in the KEGG database. A total of 116 genes were found to be statistically significant, however, only 54 of them could be identified and were distributed into different biologically relevant functions (Table S2).

Hierarchical clustering of the correlation data matrix between genes and taxonomy at the gene level revealed the presence of two highly populated clusters (Figure 3A). Remarkably, except for *Lachnospiraceae bacterium* 5_1_63FAA, those species that increased in abundance after the intervention and were positively associated with GLPs response (*Clostridium* sp. CAG:75, *Clostridium* sp. CAG:127, *Clostridium* sp. CAG: 230) had similar gene association patterns. These patterns contrasted with those for *Mitsuokella_uc*, whose abundance decreased at follow‐up and inversely correlated with GLP response. Only a minor fraction of the total studied genes (12%, seven genes) could be projected onto a metabolic map (Figure 3B), indicating that most of the altered genes have other biological functions. Indeed, no particular pathway was enriched, suggesting a global modulation of gut microbiota in terms of metabolism.

**FIGURE 3:**
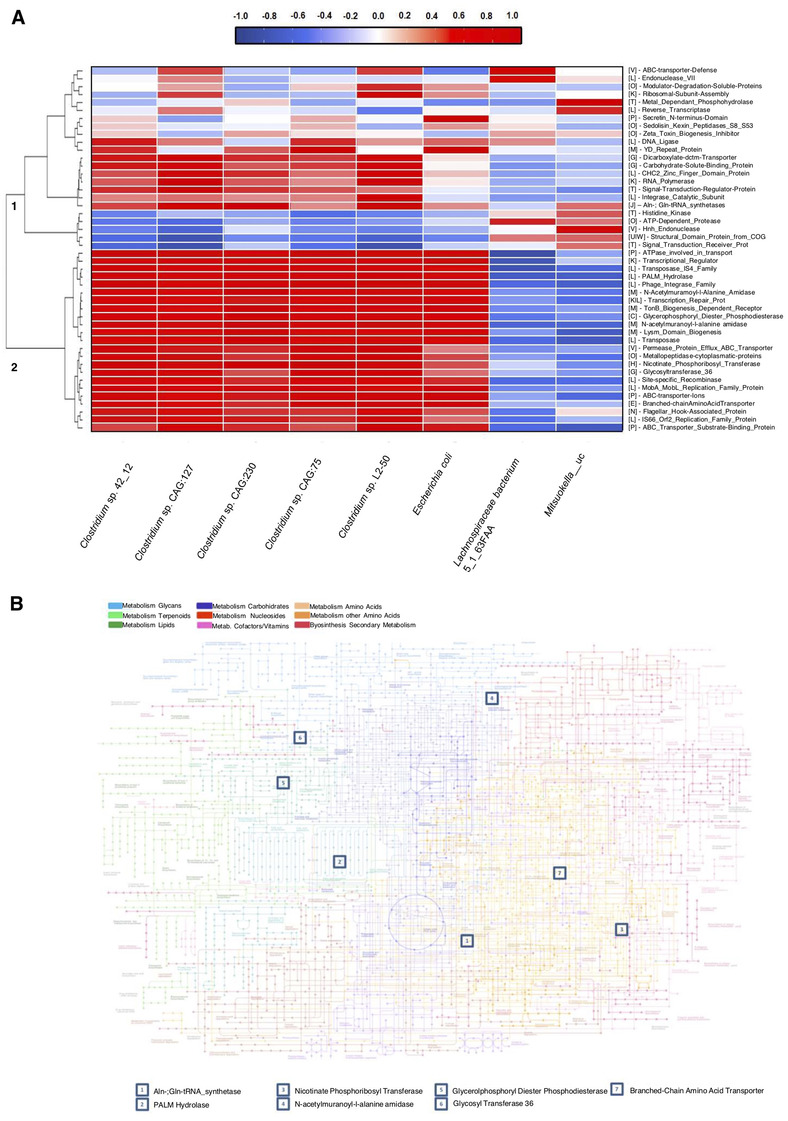
Spearman correlation analysis of microbiota species and metabolic bacterial function for a set of 45 genes. (A) Hierarchical clustering of the correlation data matrix was conducted at the gene level, which revealed the presence of two highly populated clusters (labelled as 1 and 2). Annotation of metabolic pathways according to the KEGG database, sorted in alphabetical order: [C]: energy production and conversion; [E]: amino acid transport and metabolism; [G]: carbohydrate transport and metabolism; [H]: coenzyme transport and metabolism; [J]: translation; ribosomal structure and biogenesis; [K]: transcription; [L]: replication, recombination, and repair; [M]: cell wall/membrane/envelope biogenesis; [O]: post‐translational modification; protein turnover, chaperones; [P]: inorganic ion transport and metabolism; [T]: signal transduction mechanism; [U|W]: intracellular trafficking and vesicular transport; [V]: defence mechanism. Correlations where an associated *p*‐adjusted value was greater than 0.25 were neglected for additional analysis. Genes associated with replication, recombination, and repair (encoded as [L]), such as MobA/MobB, site‐specific recombinases, integrases, and transposases, were found to positively correlate with an increase in the presence of *Clostridium* species. A similar pattern was found (with an equivalent number of enzymes, 4) with post‐translational modification enzymes ([O]) and enzymes involved in signal transduction ([T]). An increase in the abundance of the metabolic enzymes glycosyltransferase_36, dicarboxylate transporter, and carbohydrate‐solute binding proteins were positively correlated with Clostridiaceae family members. Genes associated with amino‐acid transport and metabolism ([E]) and inorganic ion transport ([P]), such as branched‐amino acid transporter and ABC‐related transporter, were also modified. (B) Gene projection of statistically relevant enzyme subset on a KEGG‐modified metabolic enzyme pathway. This analysis revealed only a minor fraction of the total studied genes (12%, seven genes) that could be projected into a metabolic map constellation:Aln‐; Gln‐t‐RNA synthase ([J]), PALM hydrolase ([L]), nicotinate‐phosphoribosyltransferase ([H]), N‐acetylmuranoyl‐l‐alanine amidase ([M]), glycerol‐phosphoryl‐diester‐phospho‐diesterase ([C]), glycosyltransferase 36 ([G]), and branched‐amino acid transporter ([E]). All correspond to different biological pathways

Our results lead us to consider that while diet‐induced weight loss impacts gut microbiota composition, and the global metabolic regulation of microbial community interactions maintains a balanced ecosystem. This would agree with the plasticity of gut microbiota, allowing rapid adaption to environmental changes. In conclusion, our study shows that GLP secretion, which is dependent on body weight and metabolic status, is linked to specific gut microbiota. Further mechanistic studies are warranted to understand how gut microbiota regulate GLP secretion and whether those bacterial strains associated with GLP dynamics might represent novel probiotic approaches to restore host mucosal GLP response in obesity.

## CONFLICT OF INTEREST

The authors declare no conflict of interest.

## ETHICS APPROVAL AND CONSENT TO PARTICIPATE

All participants gave their informed consent, and the study was reviewed and approved by the ethics and research committee of University Hospital Joan XXIII from Tarragona, Spain.

## AUTHOR CONTRIBUTIONS

M‐Mar Rodríguez‐Peña, Gemma Llauradó, and Brenno Astiarraga participated in the sample processing, functional studies, laboratory determinations, and statistical analysis. M‐Mar Rodríguez‐Peña, Jesus Seco, Brenno Astiarraga, and Victoria Ceperuelo‐Mallafré wrote the manuscript. Jesus Seco, Adrià Caballé, Camille Stephan‐Otto Attolini, and Vicente Pérez‐Brocal performed statistical analysis of taxonomical and clinical features. Andrés Moya and Ana Megía provided scientific discussion and revised the manuscript. Silvia Pellitero and Núria Vilarrasa participated in the human sample recruitment and conducted the clinical trial. Joan Vendrell and Sonia Fernández‐Veledo conceived, designed, and supervised the research project and wrote the manuscript. Joan Vendrell and Sonia Fernández‐Veledo are also the guarantors of this work and, as such, had full access to all the data in the study and take responsibility for the integrity of the data and the accuracy of the data analysis.

## FUNDING INFORMATION

Instituto de Salud Carlos III, Grant Numbers: PI14/00228, PI17/0153 and PI20/00338; Ministerio de Ciencia e Innovación, Grant Numbers: RTI2018‐093919‐B‐I00 and PID2019‐105969GB‐I00; Generalitat Valenciana, Grant Number: PROMETEO/2018/A/133.

## Supporting information

Supplementary information

Supplementary information

TableS2

## Data Availability

All data generated or analyzed during this study are included in this published article and its Supplementary Information. Further information and requests for resources and reagents should be directed to and will be fulfilled by the corresponding authors. The metagenome data sets from this study are available in the EBI Short Read Archive under the study accession PRJEB43147, with accession numbers [ERX5107074 – ERX5107109].
