# Supplementary material for: Changes in glucagon‐like peptide 1 and 2 levels in people with obesity after a diet‐induced weight‐loss intervention are related to a specific microbiota signature: A prospective cohort study
Source: Clin Transl Med. 2021 Nov 6;11(11):e575. doi: 10.1002/ctm2.575 (PMC8571947; doi:10.1002/ctm2.575)
Supplement: Supplementary file 1 — Supplementary information [file CTM2-11-e575-s002.docx]

**SUPPLEMENTARY INFORMATION**

**METHODS**

**Patients**

Twenty-six non-morbid subjects with obesity, with a body mass index (BMI) range ≧30 ≦40 kg/m^2^ were invited to participate in the setting of a prospective interventional non-randomized study (<https://doi.org/10.1186/ISRCTN12973246>). Inclusion criteria were age >18 and <65 years, absence of acute or chronic systemic disease other than obesity, absence of any pharmacological treatment, and weight stability for at least 3 months prior to the study. The study was performed at the University Hospital Joan XXIII from Tarragona (Spain) started in June 2016 and ended in June 2017. All participants received a low-calorie Mediterranean-type diet (20 kcal/kg baseline body weight) comprising 4 daily meals for 6 months (50% carbohydrates, 25–30% lipids and 20–25% protein) and were encouraged to increase their physical activity. Monthly visits were scheduled with a dietician and included a health status review and diet reminder recommendations. An effective weight loss was established when the subject lost at least 10% of their initial weight at the end of the follow-up. Only subjects that achieved this weight target were considered for this study (Supplementary Figure 4).

The study was conducted according to the principles of the Declaration of Helsinki and was approved by the corresponding local ethics committee. All subjects signed an informed consent before entry into the study. We used the STROBE cohort reporting guidelines ^1^.

**Anthropometry and functional studies**

Body weight and height, waist circumference and blood pressure were measured at each visit. A meal tolerance test (MTT) was conducted at baseline and at the end of the 6-monthfollow-up. The MTT was performed in the morning after an overnight fast, with no food or drink (except for water) after 8 P.M. the preceding day. An intravenous line was established in the ante cubital vein for venous blood sampling. Patients ingested a standardized liquid meal beverage (16% proteins, 49% carbohydrates, and 30% lipids [320 kcal]; Iso-source®, Nestle Health Science) over 5 min. Blood was sampled before meal ingestion (time 0 min) and at 15, 30, 60, and 120 min after meal ingestion ^2^.

**Laboratory determinations**

Whole blood was stored at 4°C and serum/plasma was separated and immediately frozen at -80 °C. Serum glucose, total cholesterol, high-density lipoprotein cholesterol (HDLc), triglycerides, aspartate transaminase (AST), alanine aminotransferase (ALT) and gamma-glutamyltransferase (GGT) were measured by standard enzymatic methods(ADVIA Centaur, Siemens Healthcare, Erlangen, Germany and Analox GM-9, London, UK). Low-density lipoprotein cholesterol levels (LDLc) were calculated using the Friedewald equation ^3^. Plasma insulin and C-peptide levels were determined with immunochemiluminometric assays (ADVIA Centaur). Fasting GLP-1 and GLP-2 levels were analyzed by enzyme-linked immune sorbent assay (EZGLP1T-36K and EZGLP2-37K,respectively; Merck KGaA, Darmstadt, Germany) ^2^. Circulating serum zonulin levels were assessed using the enzyme-linked immune sorbent assay (MY Biosource, San Diego, CA) ^4–6^.

**Data analysis of metabolic parameters**

GPL-1- and GLP-2-response were defined as the area under the time concentration curve (AUC) for the GLP-1/2 metrics between times 0 to time 60 min. For every individual, these areas were estimated considering 4 measurements obtained at times 0, 15, 30, and 60 min using the AUC function from the DescTools R package, under the geometric trapezoid approach. Other geometric approaches were tested (spline- and stepwise-based) with comparable results.

Mean plasma values were calculated as the ratio between the AUC by the period of the test in minutes. The homeostasis model assessment of insulin resistance (HOMA-IR) index was calculated as the product of the fasting insulin level (mIU/L) and the fasting glucose level (mg/dL) divided by 405.

**Stool sample collection, DNA extraction, and metagenomic sequencing**

To assess taxonomic and functional changes of fecal samples collected at baseline and six months after the dietary intervention we used shotgun sequencing of stool DNA for whole metagenome analysis. Patients collected fresh stool samples at home, which were immediately frozen in their home freezer at -20ºC. Frozen samples were delivered to the hospital within 2 days using insulating polystyrene foam containers and were kept at -80ºC until analysis. DNA extraction was performed using the QIAamp DNA stool kit (Qiagen, Hilden, Germany). DNA quantification was performed using a Qubit 3.0 Fluorometer (Thermo Fisher Scientific, Carlsbad, CA), and 1 ng of each sample (0.2 ng/μL) was used for shotgun library preparation using the Nextera XT DNA Library Preparation Kit (Illumina, Inc., San Diego, CA). Sequencing was carried out on a NextSeq 500 sequencer (Illumina) with 150-bp paired-end chemistry, at the Sequencing and Bioinformatic Service of FISABIO (Valencia, Spain).

Metagenomics sequencing can assign both taxonomic and functional annotations to the total DNA reads. Taxonomic assignments were performed withKaiju ^7^, a program for computationally efficient and sensitive taxonomic classification of high-throughput sequencing reads from metagenomics sequencing experiments. Each sequencing read was assigned to a taxon in the NCBI taxonomy (<https://www.ncbi.nlm.nih.gov/taxonomy>) by comparing it to the microbial subset of the NCBI BLAST non-redundant database, not including fungi and microbial eukaryotes. Genera for which the resolution at species level could not be reached were annotated with the _uc appendix (meaning unclassified). This implies that of two or more species showed similar scores, and therefore a best hit could not be unambiguously assigned. Therefore, their analysis correspond to the genus level.

To assign functional annotations, the reads were first assembled into contigs using Ray software (v2.3.1)^8^. Prodigal software (v2.6.3) ^9^ was deployed to identify genes inside contigs, and functional annotation of the genes against the Kyoto Encyclopedia of Genes and Genomes (KEGG) database ^10^ was performed with HMMER (v3.1b2) ^7^. The abundance of the annotated genes was finally measured by counting aligned reads with megaBLAST (v2.2.26) ^11^, only considering 97% identity over the query coverage (100%). A total of 116 genes were found to be statistically significant, however, only after Bonferroni correction with a p-adjusted value < 0.05. After removal of poorly characterized or partially unknown genes, a subset of 54 genes was retained.

**Statistical analysis of taxonomical and clinical features**

Sequence data were analyzed using the phyloseqR (version 1.28.0) ^12^, vegan(version 2.5-5) ^13^, metagenomeSeq (version 1.26.2) ^14^ and ggplot2 packages implemented in R. Taxonomical analysis reached species level if possible unless otherwise stated (_uc annotated). Before data normalization, singletons and other rare species (represented by reads of low abundance, <0.01%) were removed to minimize the number of potentially erroneous sequences in the analysis.

Abundance raw-data counts were normalized using the cumulative sum scaling (CSS) method ^14^. The zero-inflated Gaussian mixture model (FitZig), was applied over the cumulative sum scaling-normalized data to account for abundance differences of species between pre-and post-treatment, by including the patient identifier variable (IDPAT) as a covariate in the analysis to account for the paired nature of the data. Briefly, to accurately determine taxonomic counts of sparse data, this statistical method generates a modelled count distribution as a mixture of two distinguished distributions, consisting of a point mass at zero and a normal distribution. The zero counts are modelled with the former distribution, and the remaining counts are log-transformed and modelled based on their normality data (latter distribution).The fitted statistical model is generated upon parameters that are estimated with a maximization-expectation algorithm coupled to a moderated statistical-t/test. This approach has proven to be more effective than comparable differential abundance methods such as DESeq, edgeR or Voom ^15^.

To evaluate alpha diversity of bacterial communities, Shannon’s index and OTUs (Observed species) were calculated using the phyloseq R package. The proportion and composition of the most abundant species in the data was aggregated at both Phylum and Family levels. The beta diversity was computed under the Bray-Curtis dissimilarity index ^16^, and linked to clinical variables using the distance-based Adonis procedure ^17^, implemented in the. Principal component analysis (PCA) on the CSS normalized data was applied to represent the percentage of explained data variation in the most relevant clinical variables, as well as to identify potential outliers and rare species. Clinical variables were tested for normality using the Shapiro-Wilk test before running inferential statistics. Non-parametric data were evaluated by the Wilcoxon rank-sum test, while normally distributed variables were examined by Student’s t-test. P-values less than 0.05 were considered significant after applying the Benjamini-Hochberg multiple testing procedure.The relationship between clinical variables and alpha diversity measures was evaluated using a linear mixed effects model, considering the alpha diversity as response variable, the clinical variable as fixed effect and the patient as random effect Spearman’s rank correlation was used to investigate associations between microbial data, and reported clinical variables using a customized z-score metric supported by a global signature correction approach ^18–20^. For correlational studies all data (basal and 6 months) are included in the analysis.

1. Von Elm E, Altman DG, Egger M, Pocock SJ, Gøtzsche PC, Vandenbroucke JP. Declaración de la iniciativa STROBE (Strengthening the Reporting of Observational Studies in Epidemiology): Directrices para la comunicación de estudios observacionales. *Rev Esp Salud Publica*. 2008;82(3):251-259. doi:10.1157/13119325

2. Astiarraga B, Martínez L, Ceperuelo-Mallafré V, et al. Impaired succinate response to a mixed meal in obesity and type 2 diabetes is normalized after metabolic surgery. *Diabetes Care*. 2020;43(10):2581-2587. doi:10.2337/dc20-0460

3. Friedewald WT, Levy RI, Fredrickson DS. Estimation of the concentration of low-density lipoprotein cholesterol in plasma, without use of the preparative ultracentrifuge. *Clin Chem*. 1972;18(6):499-502. doi:10.1093/clinchem/18.6.499

4. Serena C, Ceperuelo-Mallafré V, Keiran N, et al. Elevated circulating levels of succinate in human obesity are linked to specific gut microbiota. *ISME J*. 2018;12(7):1642-1657. doi:10.1038/s41396-018-0068-2

5. Smecuol E, Sugai E, Niveloni S, et al. Permeability, zonulin production, and enteropathy in dermatitis herpetiformis. *Clin Gastroenterol Hepatol*. 2005;3(4):335-341. doi:10.1016/S1542-3565(04)00778-5

6. Wang W. *Zonulin as Modulator of Intestinal Tight Junctions*.; 2000.

7. Menzel P, Ng KL, Krogh A. Fast and sensitive taxonomic classification for metagenomics with Kaiju. *Nat Commun*. 2016;7. doi:10.1038/ncomms11257

8. Boisvert S, Raymond F, Godzaridis É, Laviolette F, Corbeil J. Ray Meta: Scalable de novo metagenome assembly and profiling. *Genome Biol*. 2012;13(12). doi:10.1186/gb-2012-13-12-r122

9. Hyatt D, Chen GL, LoCascio PF, Land ML, Larimer FW, Hauser LJ. Prodigal: Prokaryotic gene recognition and translation initiation site identification. *BMC Bioinformatics*. 2010;11. doi:10.1186/1471-2105-11-119

10. Kanehisa M, Araki M, Goto S, et al. KEGG for linking genomes to life and the environment. *Nucleic Acids Res*. 2008;36(SUPPL. 1). doi:10.1093/nar/gkm882

11. Altschul SF, Gish W, Miller W, Myers EW, Lipman DJ. Basic local alignment search tool. *J Mol Biol*. 1990;215(3):403-410. doi:10.1016/S0022-2836(05)80360-2

12. McMurdie PJ, Holmes S. Phyloseq: An R Package for Reproducible Interactive Analysis and Graphics of Microbiome Census Data. *PLoS One*. 2013;8(4). doi:10.1371/journal.pone.0061217

13. Jari Oksanen: Vegan: community ecology package - Google Scholar.

14. Paulson JN, Colin Stine O, Bravo HC, Pop M. Differential abundance analysis for microbial marker-gene surveys. *Nat Methods*. 2013;10(12):1200-1202. doi:10.1038/nmeth.2658

15. Weiss S, Xu ZZ, Peddada S, et al. Normalization and microbial differential abundance strategies depend upon data characteristics. *Microbiome*. 2017;5(1):1-18. doi:10.1186/s40168-017-0237-y

16. Bray JR, Curtis JT. An Ordination of the Upland Forest Communities of Southern Wisconsin. *Ecol Monogr*. 1957;27(4):325-349. doi:10.2307/1942268

17. Anderson MJ. A new method for non-parametric multivariate analysis of variance. *Austral Ecol*. 2001;26(1):32-46. doi:10.1111/j.1442-9993.2001.01070.pp.x

18. Lee E, Chuang HY, Kim JW, Ideker T, Lee D. Inferring pathway activity toward precise disease classification. *PLoS Comput Biol*. 2008;4(11). doi:10.1371/journal.pcbi.1000217

19. Efron B, Tibshirani R. On testing the significance of sets of genes. *Ann Appl Stat*. 2007;1(1):107-129. doi:10.1214/07-aoas101

20. Mestres AC, Llergo AB, Attolini CSO. Adjusting for systematic technical biases in risk assessment of gene signatures in transcriptomic cancer cohorts. *bioRxiv*. Published online July 2018. doi:10.1101/360495
