# Supplementary material for: Changes in glucagon‐like peptide 1 and 2 levels in people with obesity after a diet‐induced weight‐loss intervention are related to a specific microbiota signature: A prospective cohort study
Source: Clin Transl Med. 2021 Nov 6;11(11):e575. doi: 10.1002/ctm2.575 (PMC8571947; doi:10.1002/ctm2.575)
Supplement: Supplementary file 2 — Supplementary information [file CTM2-11-e575-s003.pdf]

Supplementary Table 1. Main anthropometric and metabolic variables.

Variables	Baseline	Follow-up 6 months	p-value
**Sex (male/female), *n***	6/12	6/12	----
**Age (years)**	48.2 ± 11.6	48.7 ±11.5	ns
**BMI (kg/m^2)**	35.4 ± 3.9	31.4 ± 3.6	**<0.0001**
**Waist (cm)**	108.8 ± 8.9	100.3 ± 9.3	**<0.0001**
**Fasting glucose (mmol/L)**	5.2 ± 0.6	4.5 ± 0.4	**0.002**
**Fasting insulin (pmol/L)**	104.6 ± 34.3	42.8 ± 16.9	**0.009**
**Fasting C-peptide (nmol/L)**	0.59 ± 0.21	0.44 ± 0.14	**0.002**
**HOMA-IR**	4.0 ± 1.4	2.0 ± 1.2	**0.006**
**HbA$_{1c}$ (mmol/mol)**	36 ± 4	32 ± 4	**0.027**
**Urates (mg/dl)**	5.6 ± 1.2	5.3 ± 1.4	ns
**Total cholesterol (mmol/L)**	4.96 ± 0.63	4.47 ± 0.66	**0.019**
**HDL-cholesterol (mmol/L)**	1.23 (1.12 - 1.57)	1.26 (1.03 – 1.38)	ns
**LDL-cholesterol (mmol/L)**	3.06 ± 0.46	2.71 ± 0.47	**0.013**
**Triglycerides (mmol/L)**	1.08 (0.9 – 1.34)	1.02 (0.7 – 1.16)	ns
**ALT (U/L)**	21.1 (16.2 – 23.0)	20.5 (18.5 – 22.7)	ns
**AST (U/L)**	26.1 (17.0 – 31.4)	17.2 (16.0 – 21.3)	**0.014**
**GGT (U/L)**	18.3 (11.5 – 25.2)	13.4 (10.6 – 17.2)	**0.001**
**Fasting GLP-1 (pmol/L)**	44.4 ± 16.8	33.0 ± 11.0	**0.023**
**GLP-1 response (AUC)***	461.1 ± 92.3	604.7 ± 97.3	ns
**Fasting GLP-2 (ng/mL)**	4.4 ± 1.3	3.4 ± 1.0	**0.001**
**GLP-2 response (AUC)***	18.7 ± 4.0	25.1 ± 5.5	ns
**Fasting Zonulin (ng/mL)**	115.3 ± 59.4	120.3 ± 37.2	ns

Data are presented as mean ± SD or median (25th - 75th percentiles) as appropriate, based on Shapiro normality-test evaluation. p-values for the normal distributed variables were calculated using Student's paired t-test; the Wilcoxon signed rank test was used for the non-normal distributed variables. p-value <0.05 was considered significant. ns: non-significant. Abbreviations: HDL, high density lipoprotein; LDL low density lipoprotein; ALT, alanine aminotransferase; AST, aspartate aminotransferase; GGT, gamma glutamyltransferase. AUC, area under the curve.

***** A meal tolerance test (MTT) was conducted at baseline and at the end of the 6-monthfollow-up. The MTT was performed in the morning after an overnight fast, with no food or drink (except for water) after 8 P.M. the preceding day. An intravenous line was established in the ante cubital vein for venous blood sampling. Patients ingested a standardized liquid meal beverage (16% proteins, 49% carbohydrates, and 30% lipids [320 kcal]; Iso-source, Nestle Health Science) over 5 min. Blood was sampled before meal ingestion (time 0 min) and at 15, 30, 60, and 120 min after meal ingestion

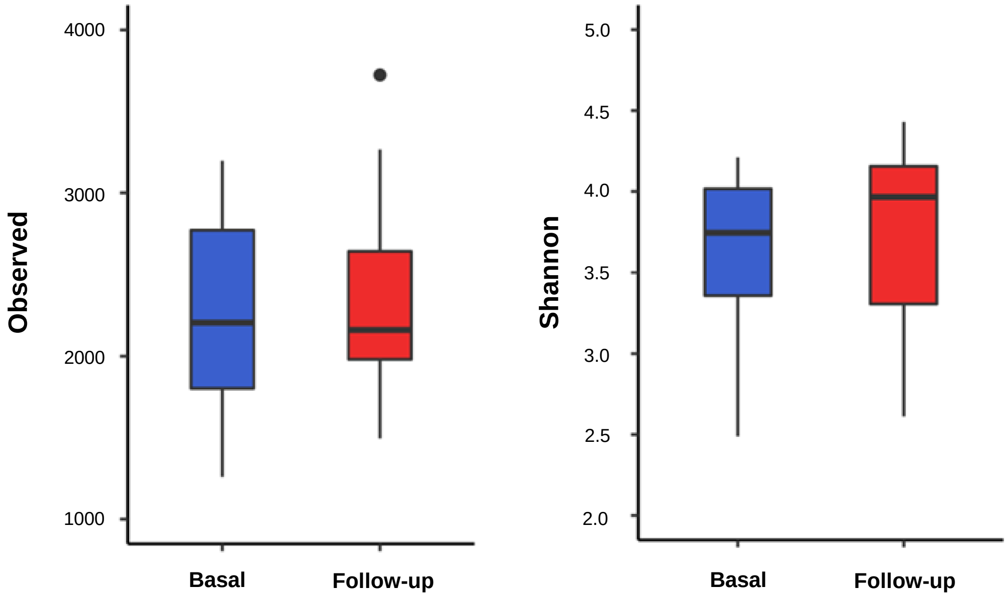

Supplementary Figure 1. Box plot representation of the observed OTU number (measuring species richness) and the Shannon index diversity (measuring richness and evenness) vs diet. The dietary intervention had no significant impact on the richness and Shannon diversity.

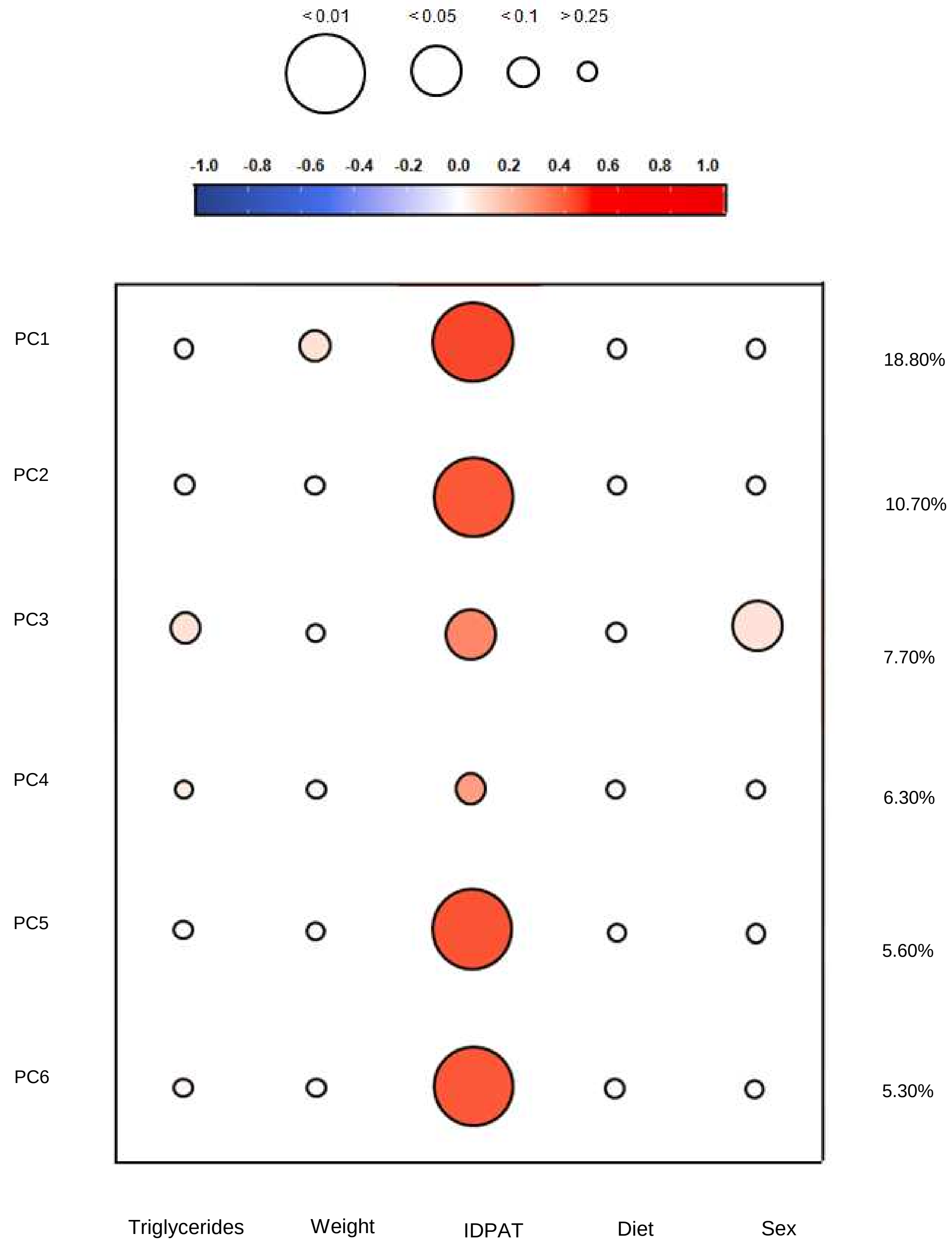

Supplementary Figure 2. Principal components association (Abundance data analysis) with condition and normalized (and trimmed for rare cases) data. IDPAT variable correlates with all principal components tested so this variable can be seen as one of the main sources driving variability in the data matrix.

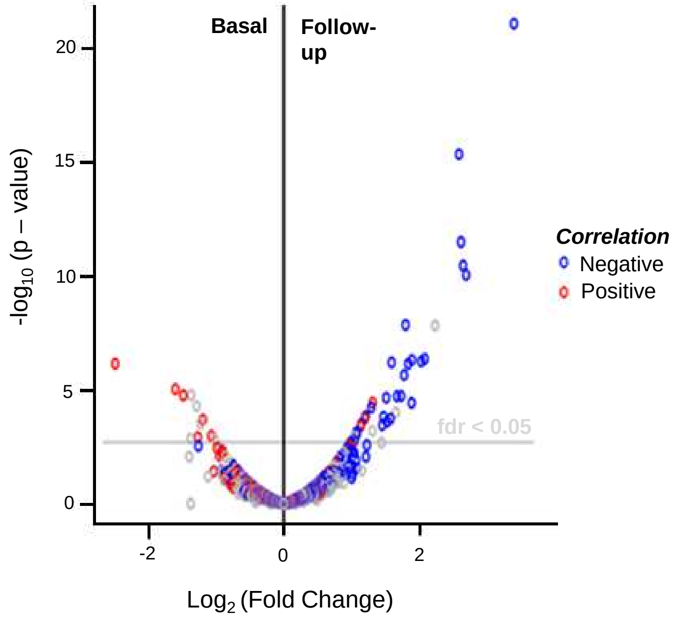

Supplementary Figure 3. Volcano plot depicting differential abundant operational taxonomic units (OTUs) for basal and 6 months follow-up (log2 fdr<0.05). The bacteria represented in blue showed a negative correlation while the bacteria represented in red showed a positive correlation with clinical and analytical parameters. Differential abundance analysis in the elapsed studied time revealed more than 40 species or genus with a significant variation in terms of fold-change and statistical relevance when considered simultaneously. Remarkably, most of the bacteria overrepresented after weight loss (log2false discovery rate <0.05) showed a negative correlation with clinical and analytical parameters.

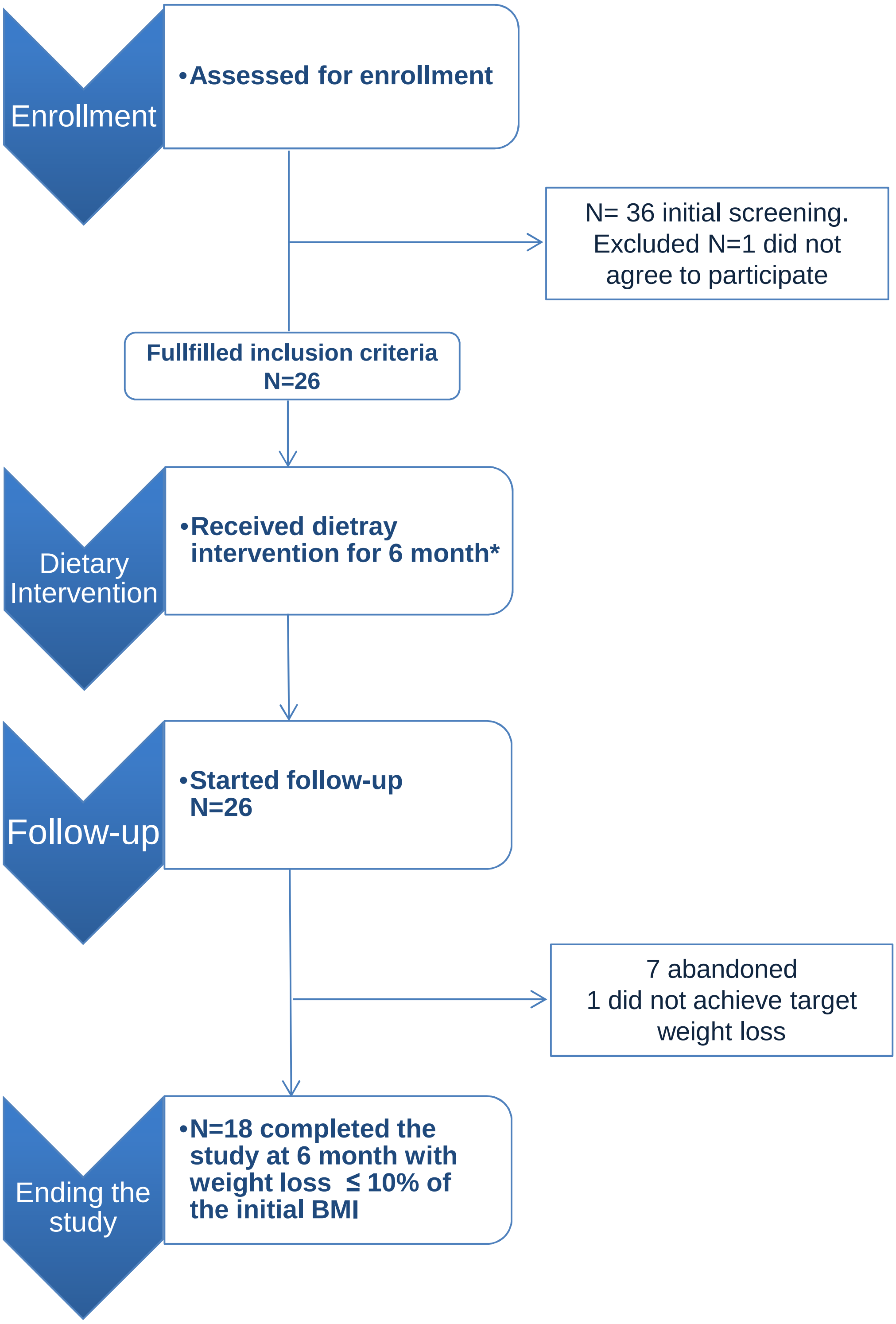

Supplementary Figure 4. Study flowchart.
*All participants underwent a low-calorie Mediterranean-type Diet (20 Kcal/Kg baseline body weight), with four daily meals for six months (50% carbohydrates, 25- 30% lipids, and 20 to 25% protein). They were encouraged to increase their physical activity. Monthly visits were scheduled with a dietician and included a health status review and diet reminder recommendations. Meal tolerance test; Laboratory determinations; Stool sample collection to metagenomic sequencing were performed at the beginning and the end of the follow-up.
